# Dynamic Manipulation Skill Learning for Tactile Myoelectric Prosthetic Hands in Tool Handling

**DOI:** 10.34133/cbsystems.0572

**Published:** 2026-05-13

**Authors:** Boao Li, Shuhui Wu, Ting You, Shixian Wang, Ziming Chen, Ye Liu, Di Guo, Fuchun Sun, Guangyuan Xu, Du Jiang, Gongfa Li, Bin Fang

**Affiliations:** ^1^ Key Laboratory of Metallurgical Equipment and Control Technology of Ministry of Education, Wuhan University of Science and Technology, Wuhan 430081, China.; ^2^ School of Artificial Intelligence, Beijing University of Posts and Telecommunications, Beijing 100080, China.; ^3^ Beijing Sport University, Beijing 100084, China.; ^4^ Guangdong Institute of Intelligence Science and Technology, Guangdong 519031, China.; ^5^ Institute for Artificial Intelligence, State Key Lab of Intelligent Technology and Systems, Tsinghua University, Beijing 100084, China.; ^6^ Institute of Embodied AI, Wuhan University of Science and Technology, Wuhan 430081,China.

## Abstract

Continuous tool operation with a myoelectric prosthetic hand is considerably more complex than discrete grasping tasks. This complexity arises because the control system must maintain stable, adaptive, and coordinated motions under varying loads and unpredictable interactions. In human motor control, this stability is achieved through a biological sensorimotor closed loop, where tactile feedback continuously modulates neural signals to adapt to environmental changes. Inspired by these mechanisms for reducing grasp instability caused by external shocks, this study designed a multimodal controller termed the tactile, kinesthetic, and electromyography (EMG) bionic gripping controller (TKE-BGC). It integrates tactile, kinematic, and EMG information. Initially, multimodal data—encompassing tactile signals, joint angles, and EMG patterns—were collected from able-bodied users during tool manipulation via a data glove. Subsequently, the TKE-BGC model was trained on these data, utilizing a Transformer encoder to extract high-level features and a multilayer perceptron to predict joint angles in real time. Based on this controller, this paper presents a prosthetic control framework developed through human skill transfer. Unlike conventional fixed force or force follows strategies that struggle with dynamic impacts or tracking delays, this framework enables robust end-to-end adaptive control. Tested across 4 seen and unseen tool operation tasks, the proposed method demonstrated precise detailed performance. Specifically, it significantly reduced the number of tool drops and shortened task completion times compared to the baseline methods. Furthermore, it achieved human-like average contact forces and substantially lowered the user’s physical workload, requiring noticeably less muscle effort than the force follows strategy (e.g., average EMG amplitude, 0.0023 versus 0.0124). By rapidly adjusting grip force through feedback and effectively mitigating instability, this research holds significant practical value in enhancing the daily independence of amputees and supporting their vocational rehabilitation and reemployment.

## Introduction

Upper limb amputation results in the loss of grasping and manipulation capabilities, severely affecting amputees’ ability to perform daily activities. Myoelectric prosthetic hands interpret surface electromyography (sEMG) signals to infer users’ motion intentions and subsequently drive the prosthesis to execute corresponding actions, serving as an important approach for restoring hand function in amputees. With the advancement of control strategies, prosthetic hand control has evolved from the early on–off control to proportional control [1–3], enabling basic regulation of a limited number of degrees of freedom (DoFs). Furthermore, by applying advanced signal processing techniques such as pattern recognition (PR) [4–9] and continuous regression [10–13], researchers have achieved coordinated control of multiple DoFs, demonstrating superior performance compared to conventional proportional control in supervised home-use environments [14].

Currently, research on prosthetic hands mainly focuses on achieving efficient and stable grasping of various objects [15–22]. Chen et al. [23] proposed a multistage grasping control method, which effectively improves grasping efficiency through a one-hand multigrasp strategy. Xu et al. [24] developed a vision-based prosthetic hand control approach that enables efficient autonomous grasping relying solely on external visual input, thereby significantly reducing the users’ grasping workload. These studies have substantially improved prosthetic grasping performance. However, functional restoration should extend beyond simple object holding to more valuable and complex manipulation tasks.

Some studies have addressed basic manipulation tasks, such as object placement and sorting [25,26], as well as activities of daily living, including squeezing toothpaste [23], tying shoelaces [27], pouring water [28], organizing luggage, and cutting vegetables [29]. Although these tasks contribute to improving amputees’ functional independence, they remain insufficient for tool-based operations that require precise force control, sustained application, and dynamic stability, such as hammering, sawing, or peeling. These activities often involve strong dynamic disturbances, including rigid impacts and variable loads, which can easily cause unstable grasping or tool slippage. Conventional control methods struggle to manage these challenges, limiting the practical use of prosthetic hands in real-world work settings and reducing amputees’ opportunities to rejoin the workforce.

In contrast, humans can effectively perform complex tool operations by modulating neural signals via sensory feedback to regulate forces in real time. To emulate this adaptive capability in prosthetic hands, a biomimetic approach necessitates the transfer of these human manipulation skills. Currently, there are 2 main categories of skill transfer methods: reinforcement learning (RL) and imitation learning (IL). RL-based approaches have been applied to prosthetic and robotic control to learn grasping and manipulation strategies [30–34]. While RL can optimize control policies, it often suffers from long training times, instability in long-duration tasks, and safety concerns during real-world deployment. IL aims to directly transfer human manipulation skills to artificial systems [21,24,35,36], enabling more human-like grasping and manipulation. In the robotic domain, IL has been successfully applied to tool-use tasks, such as vegetable peeling, pouring, and other contact-intensive manipulations [37–40].

These studies have advanced robotic manipulation, improving adaptability and accuracy in contact-intensive tasks through IL. In contrast, the operation of a prosthetic hand constitutes a more complex human–machine collaboration problem, as it involves neural interfacing, biosignal decoding, and real-time adaptive control. Consequently, efficiently and safely transferring human hand manipulation skills to prosthetic hand systems remains a critical research challenge.

Therefore, this work proposes a myoelectric prosthetic control framework based on the transfer of human hand skills, specifically designed for tool-use operations and focusing on dynamic manipulation tasks. The main contributions are as follows:

1. This work extends prosthetic hand control research to dynamic tool operation scenarios. Based on manipulation data from able-bodied users, we designed a biomimetic and robust controller called the tactile, kinesthetic, and EMG bionic gripping controller (TKE-BGC). The controller integrates a Transformer encoder for high-level multimodal feature extraction and a multilayer perceptron (MLP) for joint angle prediction. It effectively improves stability during rigid impacts, delayed grasping, or insufficient grip force.
2. A myoelectric prosthetic control framework based on human hand skill transfer is introduced, shifting the focus from static grasping to dynamic manipulation and providing a new perspective for the application of prosthetic hands in real-world work environments. Two types of seen tasks, hammering nails and sawing wooden strips, are designed to evaluate the feedback correction capability of the proposed framework in complex manipulation tasks. In addition, 2 types of unseen tasks, peeler operation and desktop organization, are included to assess the generalization capability of the framework in novel manipulation scenarios. Experimental validation and user surveys demonstrate the superior performance of the proposed framework across these tasks. These results provide practical guidance to support prosthetic users’ return to work.
3. The framework establishes a bioinspired approach for cross-population hand skill transfer. It systematically bridges the gap between able-bodied user demonstrations and prosthetic manipulation. By integrating tactile, kinesthetic, and EMG inputs, the controller supports human-like grasping adaptation. This enables natural and reliable tool interaction under real-world dynamic conditions.

## Methods

**Fig. 1.**
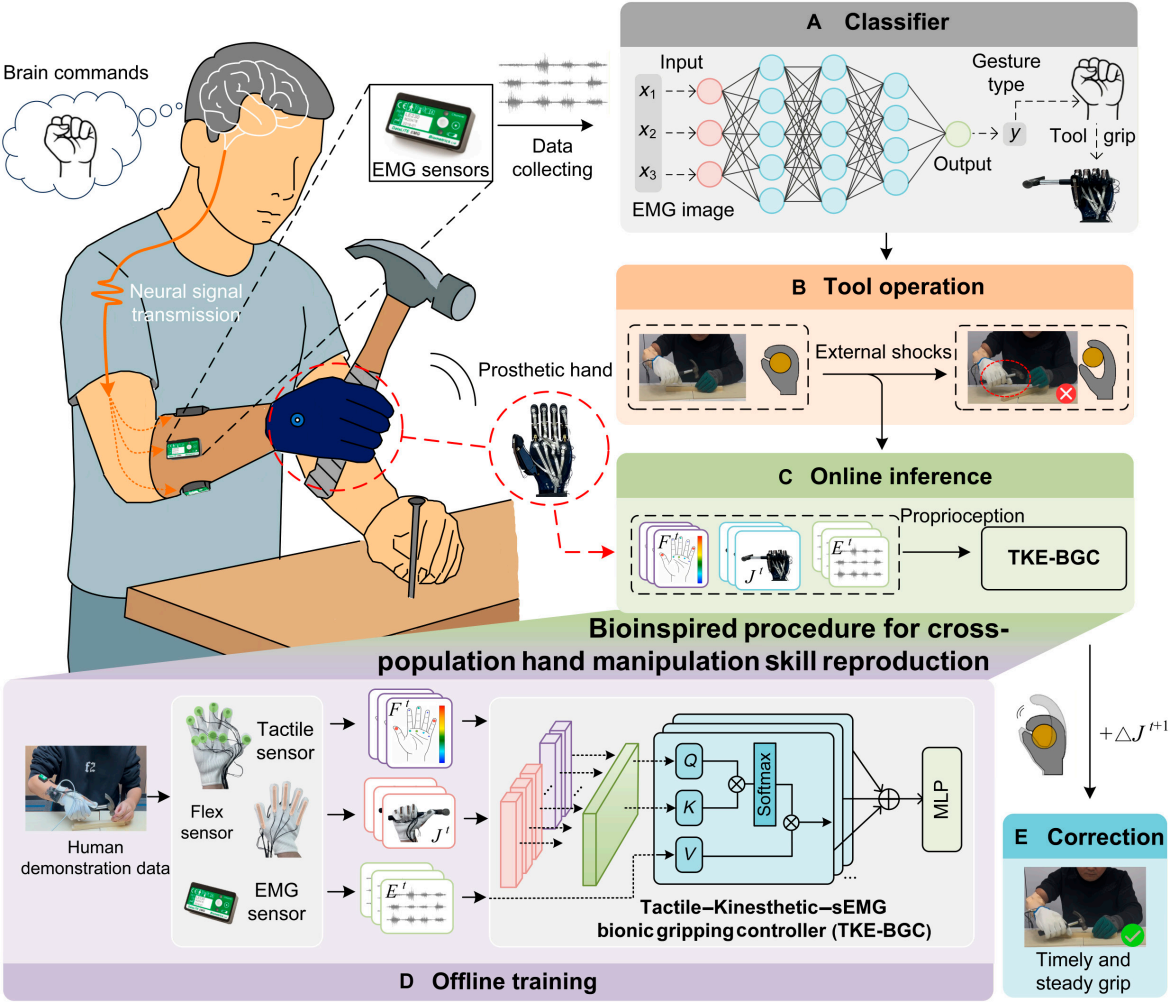
Pipeline of our framework. First, brain commands generate neural signals transmitted to the arm muscles, producing surface electromyography (sEMG) signals captured by sensors. The raw sEMG signals are collected and filtered to improve signal quality. The processed signals are input into a classifier (A) to identify gesture intent, triggering predefined prosthetic hand tool grasping commands. In dynamic tool interaction tasks, rigid impacts can easily cause prosthetic hand grasp instability (B). To address this issue, we developed a biomimetic controller (tactile, kinesthetic, and EMG bionic gripping controller [TKE-BGC]) specifically for high-dynamic scenarios (C). This controller is trained on the basis of EMG, tactile, and joint angle data generated by able-bodied individuals during tool manipulation (D). When sensors detect a risk of grasp instability, TKE-BGC immediately adjusts the joint angles using the amputee’s real-time tactile, joint angle, and EMG signals (E). This stabilizes the grasp and maintains task continuity.

The workflow of our EMG prosthetic control framework based on human skill transfer is illustrated in Fig. 1.

### Preprocessing and classifier

First, EMG signals were collected from multiple participants performing various grasping actions. The detailed experimental setup and data acquisition procedures are described in Results. The raw 3-channel sEMG signals obtained are shown in Fig. 1. To improve signal quality, the raw signals were preprocessed as follows. The sEMG signals were segmented using a 256-ms sliding window with a 128-ms step size. A second-order Butterworth band-pass filter (20 to 500 Hz) was applied to suppress low-frequency baseline drift and high-frequency noise. A 50-Hz notch filter was then used to remove power-line interference. Finally, the signals were full-wave rectified to enhance amplitude features.

The preprocessed signals were fed into an EMG PR classifier designed for dynamic operation scenarios, the DCNN-GRU model, whose detailed architecture and parameters are provided in Fig. A1 and Table A1. This model combines a dynamic convolutional neural network (DCNN) with gated recurrent units (GRUs), enabling effective extraction of the spatiotemporal features of EMG signals. It significantly reduces misclassification during dynamic movements and provides stable online recognition. Based on the classifier’s output of user grasping intentions, the system executes online EMG control of different tools through a predefined set of grasping commands.

### Acquisition of tactile, kinesthetic, and electromyographic feedback

**Fig. 2.**
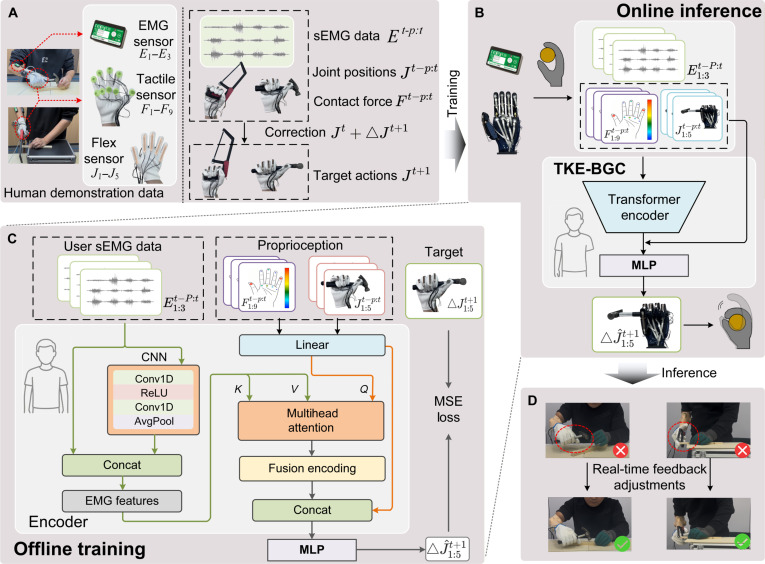
The structure of tactile, kinesthetic, and electromyography (EMG) bionic gripping controller (TKE-BGC) and imitation learning procedure. First, we collected action data from able-bodied subjects performing hammering and sawing tasks using an integrated data glove and EMG sensors (A). These data were then fed into the TKE-BGC model for training (B). The TKE-BGC input consists of *P* historical states, each containing tactile, kinesthetic, and EMG data. The TKE-BGC includes a feature Transformer encoder and a multilayer perceptron (MLP) to predict the next action increment $\Delta J_{1:5}^{t+1}$ . Specifically, (C) the myoelectric branch uses a one-dimensional convolutional neural network to extract temporal EMG features. These features are then concatenated with the original signal and fed into an attention module to preserve both local high-order features and original details, enhancing the integrity of feature representation. Kinesthetic and tactile features are linearly mapped and then fused with the EMG features using attention to capture the spatiotemporal correlations between the multiple modalities. The fused output is then concatenated with the original proprioceptive information and fed into an MLP to predict action corrections based on the current posture, ensuring control stability and physical consistency. Supervised optimization is then performed using the mean squared error (MSE) loss. CNN, convolutional neural networks; ReLU, rectified linear unit. Finally, (D) the trained model is deduced to achieve real-time stable grasping control of the prosthetic hand for amputee users.

To obtain training data based on direct human manipulation rather than robotic operation, this study integrated a data glove capable of real-time acquisition of tactile feedback and joint motion, together with EMG sensors, to enable intuitive and natural human operation, as shown in Fig. 2A. Using the data glove and EMG sensors, a kinematics-based action prediction framework can be constructed to transfer human proprioceptive skills to the prosthetic hand.

EMG signals ($E^{t}$) were acquired using a wireless EMG recording system manufactured by Biometrics Ltd., UK. The system includes a dongle receiver and 3 EMG sensors, with a sampling rate of approximately 2,000 Hz, a measurement resolution of 0.001 mV, and a range of −2 to 2 mV.

Tactile forces ($F^{t}$) were measured using a flexible tactile array integrated into the data glove. The array is fabricated on a flexible membrane substrate using force-sensitive materials, such as conductive ink and silver paste, through precise printing processes, followed by drying and curing. The tactile array can withstand a maximum normal force of approximately 20 N and has a sampling frequency exceeding 60 Hz. Each sensor was calibrated using standard weights. In this study, the measured contact force magnitude and distribution are considered a simplified yet effective representation of tactile features [41,42].

Joint positions ($J^{t}$) were captured using bend sensors embedded in the data glove. The system recorded the angles and movement directions of 5 joints at a sampling frequency above 60 Hz.

After wearing the data glove and EMG sensors, demonstrators can perform actions directly with their hands, ensuring that the operation process remains natural and intuitive. The data glove captures tactile and kinesthetic feedback in real time with minimal interference, while the EMG sensors simultaneously record muscle force changes during the operation. In addition, the glove can be moderately adapted for use on a prosthetic hand, enabling the collection of multimodal operational features in the same format as human hand demonstrations. In this study, 9 tactile sensing pads, 5 bend sensors, and 3 EMG sensors were carefully arranged and labeled. Joint angles, tactile units, and EMG channels were assigned unified codes to support consistent integration of multimodal inputs.

Overall, the data-glove-based acquisition method ensures consistent data formats across different users. This consistency allows both training and prediction to use unified input features without the need for data remapping due to structural differences in the robotic hand.

### IL based on TKE-BGC

The proposed TKE-BGC is designed to achieve stable grasp control of the prosthetic hand driven by multimodal demonstration data, as shown in Fig. 2B and C. The model input consists of 3 types of signals from a historical sequence of length P: the EMG sequence $E^{t-P:t}\in {\mathbb{R}}^{P\times N_{E}}$, the contact force sequence $F^{t-P:t}\in {\mathbb{R}}^{P\times N_{F}}$, and the joint motion sequence $J^{t-P:t}\in {\mathbb{R}}^{P\times N_{J}}$, where $N_{E}$, $N_{F}$, and $N_{J}$ denote the number of sensing channels for muscles, tactile sensors, and finger joints, respectively. The learning objective of the model is to predict the action increment $\Delta J^{t+1}=J^{t+1}-J^{t}$ at the next time step based on the current posture $J^{t}$, ensuring both motion continuity and physical consistency. The sequence length *P* is set to 20 to balance prediction accuracy and real-time performance.

First, the EMG sequence is processed by the EMG encoder branch, while the tactile and kinesthetic signals are each linearly mapped to obtain low-dimensional embeddings. As illustrated in Fig. 2C (the detailed structure of the attention module is shown in Fig. A2), in the cross-attention model, these physical embeddings act as the query, while EMG features serve as the key and value. Theoretically, this mimics the biological sensorimotor loop: The current physical state (query) actively retrieves the user’s historical motor intent (key/value). Unlike conventional feature concatenation, this mechanism acts as a dynamic contextual filter. It utilizes physical feedback to reweight noisy EMG signals, preventing EMG feature dominance and capturing a robust, EMG-driven perception–action relationship [43] against dynamic disturbances.

Specifically, the EMG branch first extracts high-level temporal features using a one-dimensional convolutional network. It is defined as follows:

$$H_{E}=\text{ConvNet}\left(E^{t-P:t}\right)\in {\mathbb{R}}^{P\times d_{E}}$$ (1)

Here, ConvNet represents a sequence of one-dimensional convolution, activation, convolution, and average pooling operations. To preserve temporal details, the raw EMG input sequence is linearly projected as follows:

$$E_{r}=E^{t-P:t}W_{r}\in {\mathbb{R}}^{P\times d_{E}}$$ (2)

The 2 features are concatenated and then linearly mapped to the dimension $d_{k}$ required by the attention mechanism:

$$\tilde{E}=\text{Concat}\left(H_{E},E_{r}\right)\in {\mathbb{R}}^{P\times 2d_{E}},\;E^{\prime }=\tilde{E}W_{r2}\in {\mathbb{R}}^{P\times d_{k}}$$ (3)

Here, $W_{r2}\in {\mathbb{R}}^{2d_{E}\times d_{k}}$ within the attention framework, and $E^{\prime }$ is mapped separately to the key and value:

$$K=E^{\prime }W_{k},\;V=E^{\prime }W_{v}$$ (4)

Here, $W_{k},W_{v}\in {\mathbb{R}}^{d_{k}\times d_{a}}$, and $d_{a}$ denotes the internal representation dimension of the single-head attention.

The kinesthetic and tactile signals are first mapped through separate linear layers into a unified query space of dimension $d_{q}$:

$$J^{\prime }=J^{t-P:t}W_{J}+b_{J},\;F^{\prime }=F^{t-P:t}W_{F}+b_{F}$$ (5)

Here, $W_{J}\in {\mathbb{R}}^{N_{J}\times d_{q}}$ and $W_{F}\in {\mathbb{R}}^{N_{F}\times d_{q}}$. The 2 are then concatenated along the feature dimension to form the raw input matrix of the query, $Q_{\text{in}}\in {\mathbb{R}}^{P\times 2d_{q}}$. To align with the dimensions of the key and value, $Q_{\text{in}}\;$ is linearly projected to obtain the query:

$$Q=Q_{\text{in}}W_{Q}\in {\mathbb{R}}^{P\times d_{a}}$$ (6)

Here, $W_{Q}\in {\mathbb{R}}^{2d_{q}\times d_{a}}$. Based on the above Q, K, and V, the multihead attention is computed as follows:

$$\text{Attn}\left(Q,\;K,V\right)=\text{softmax}\left(\frac{QK^{⊤}}{\sqrt{d_{h}}}\right)V$$ (7)

where $d_{h}=d_{a}/h$ denotes the feature dimension of each head. To enhance the model’s representation capability, we use multihead attention and apply residual connections, followed by layer normalization after each layer. The number of heads is denoted as *h*, and the resulting fusion encoding sequence is $Z\in {\mathbb{R}}^{P\times d_{Z}}$, which can be expressed as follows:

$$Z=\text{TransformerFusion}\left(Q,\;K,V\right)$$ (8)

The attention output Z captures the spatiotemporal coupling of action perception in contact forces F and joint postures J, weighted by EMG features, to ensure physical consistency. During the prediction phase, we first extract the aggregated vector representing the current time step from Z, denoted as $z_{t}=Z_{\left(t\right)}$. This vector is then concatenated with the linearly mapped current kinesthetic and tactile signals (i.e., $J^{t}W_{J_{0}}$ and $\;F^{t}W_{F_{0}}$, where $W_{J_{0}}$ and $W_{F_{0}}$ are independent projection matrices) and fed into an MLP. Formally, the prediction input vector is defined as follows:

$$u=\text{Concat}\left(z_{t},\;J^{t}W_{J_{0}},\;F^{t}W_{F_{0}}\right)$$ (9)

The action increment is then estimated through an MLP:

$$\Delta {\hat{J}}^{\;t+1}=\mathrm{MLP}\left(u;\;\Theta_{\mathrm{MLP}}\right)$$ (10)

and computes the joint posture ${\hat{J}}^{\;t+1}=J^{t}+\Delta {\hat{J}}^{\;t+1}$ at the next time step.

During training, the mean squared error (MSE) is used as the regression loss. For a training batch with sample index *n*, it is defined as follows:

$$L_{\mathrm{MSE}}=\frac{1}{N}\sum_{n=1}^{N}{\left\|\Delta {\hat{J}}^{\left(n\right)}-\Delta J^{\left(n\right)}\right\|}_{2}^{2}$$ (11)

The entire parameter set Θ was trained end-to-end using the Adam optimizer. The TKE-BGC was implemented in PyTorch and executed on a Windows 11 system equipped with an NVIDIA GeForce RTX 4070 GPU. Table A2 summarizes the key hyperparameters and detailed training settings of the TKE-BGC, including the specific architectural configurations for the Transformer encoder and the MLP.

During online inference (Fig. 2B and C), the prosthetic control system updates the historical window $\{E^{t-P:t},F^{t-P:t},J^{t-P:t}\}$ at a fixed frequency and executes the following steps sequentially. First, K and V are computed through the ConvNet and linear layers to obtain Z. Next, the aggregated vector $z_{t}$ is concatenated with the current $J^{t}$ and $F^{t}$ after linear mapping, and the resulting vector is fed into an MLP to generate $\Delta {\hat{J}}^{\;t+1}$, which is finally sent to the low-level controller for execution. This process enables EMG to “weight” and “drive” kinesthetic and tactile decision-making—that is, EMG acts as the key/value for the F*/*J query attention. The fused semantic features are then combined with the raw perceptual inputs as the action decision input, thereby integrating EMG-guided perception-driven control with direct physical constraints. This design enhances the stability and physical consistency of user control in myoelectric prosthetic grasping.

### Experiments

#### Participants

In the experimental study, 7 able-bodied male participants were recruited, designated S1 to S7. Furthermore, 3 male participants with transradial amputations were also recruited, designated A1 to A3, and their details are summarized in Table A3. All participants were informed of the study objectives and the experimental protocol designed for data collection. Written informed consent was obtained from all participants, indicating their willingness to participate and their agreement to publish the collected data. Prosthetic hand control data from 1 of the 7 able-bodied participants were used for model training, while the remaining 6 able-bodied participants and the 3 amputees participated in the testing experiments. The study was approved by the local Ethical Committee of Biomedical Research, Beijing University of Posts and Telecommunications (BUPT-A-2025022).

#### Experimental setup

**Fig. 3.**
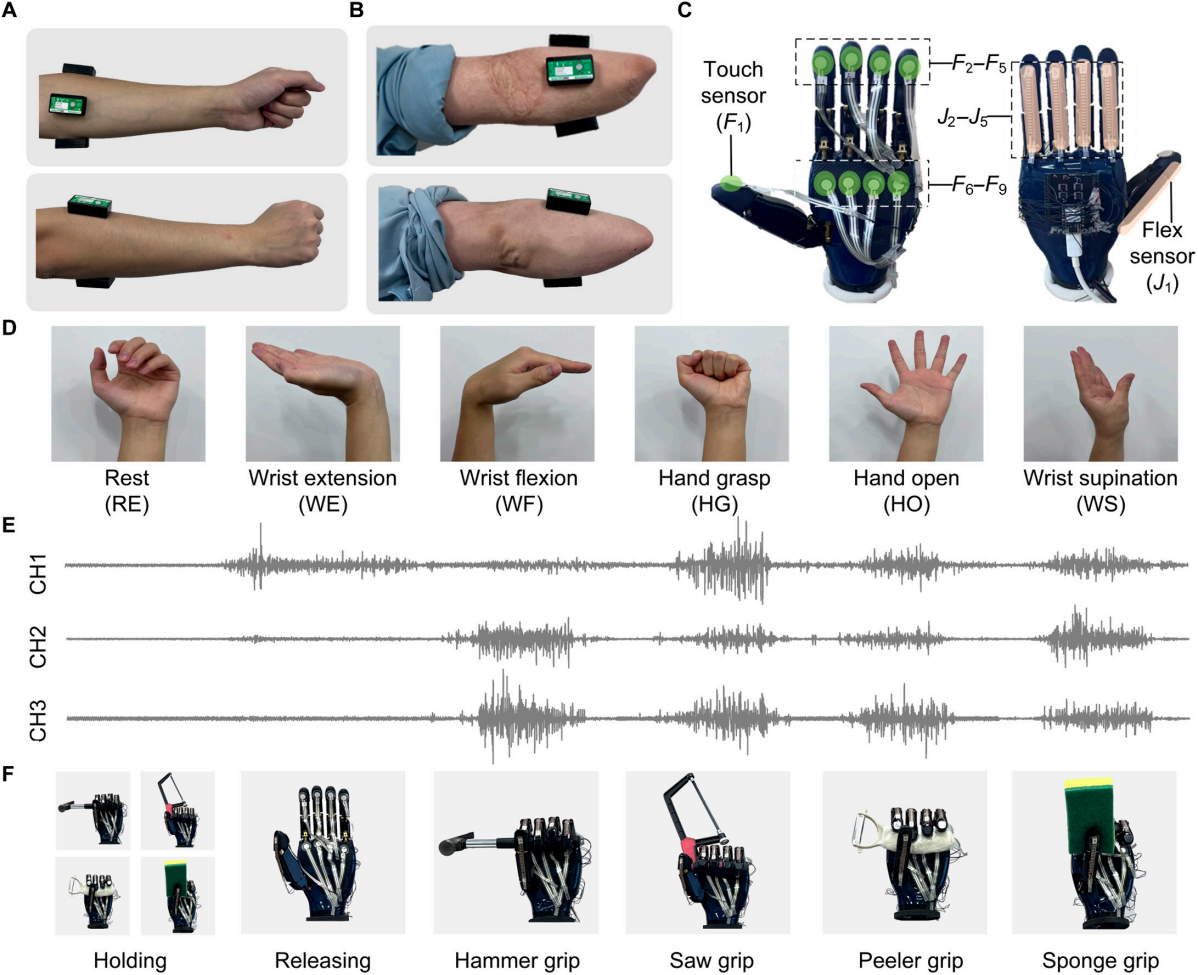
Data acquisition process. (A) Placement of electromyography (EMG) sensors on able-bodied participants and (B) on amputees. (C) Placement of tactile and bend sensors on the prosthetic hand. (D) Six gesture types for intention recognition and (E) corresponding visualizations of the raw EMG signals. CH1, channel 1. (F) Prosthetic hand tool grasping actions corresponding to each gesture.

Before the experiment, the participants’ forearm skin was cleaned with alcohol to ensure good conductivity for EMG signal acquisition. Three wireless EMG sensors were placed as shown in Fig. 3A and B, with an interelectrode distance of approximately 2 cm. The installation of tactile and joint motion sensors on the prosthetic hand is illustrated in Fig. 3C, and more technical details of the prosthetic hand can be found in [44]. Six types of hand gestures were designed for this study (Fig. 3D), including rest (RE), wrist extension (WE), wrist flexion (WF), hand grasp (HG), hand open (HO), and wrist supination (WS). The corresponding EMG signal visualizations are shown in Fig. 3E. These 6 gestures correspond to the prosthetic hand’s tool-grasping, holding, and releasing actions (Fig. 3F).

Prior to the tool manipulation experiments, the system collected data for each gesture from all participants. While 6 gestures were defined, the RE posture served as a continuous baseline state. The remaining 5 active gestures (WE, WF, HG, HO, and WS) were systematically recorded. Each active gesture involved a 10-s sustained muscle contraction, repeated 4 times per participant. In total, 10 participants × 5 gesture types × 4 repetitions yielded 200 trial-level data samples.

During model training and evaluation, these 10-s macroscopic trials were segmented into overlapping sliding windows, which served as the fundamental sample unit for the network. To ensure rigorous evaluation and completely prevent data leakage among adjacent overlapping windows, all data were randomly divided into training, validation, and testing sets at the trial level with a ratio of 6:2:2. This strategy ensures that all sliding windows derived from any single trial are strictly confined to a single subset. The offline recognition experiment results are visualized in Fig. A3A and B. Able-bodied subjects were generally able to accurately recognize the intended gestures, whereas amputee subjects exhibited higher misrecognition rates, although still within an acceptable range. The average recognition accuracies across all able-bodied and amputee subjects are summarized in Fig. A3C. The able-bodied subjects achieved an accuracy of 95.6 ± 3.1% (means ± SD), while the amputee subjects achieved 90.6 ± 5.8% (means ± SD), meeting the basic requirements for tool operation tasks.

#### Demonstration of data acquisition

This section provides a detailed description of the collection of human demonstration data, including the visualization protocol for EMG, tactile, and kinesthetic features during tool manipulation.

The experimental training dataset includes 2 types of tool operation tasks: hammer nails tasks (Fig. A4A) and sawing wood tasks (Fig. A4B). Participant performed 20 valid trials for each task. Depending on the operation type, each hammering trial lasted approximately 30 s, while each sawing trial lasted approximately 40 s. To minimize fatigue caused by continuous operation and ensure that participants remained fully relaxed before each task, a 5-min rest interval was provided between trials. Demonstration data acquisition details can be found in Movies S1 and S2.

Synchronous acquisition of data glove and EMG signals provided a foundation for capturing proprioceptive-motor integration features in multimodal tasks, thus constructing a comprehensive demonstration dataset. Fig. A4 illustrates the multimodal data visualization results obtained during the hammering and sawing tasks. Based on the collected kinematic, tactile, and EMG features, the hand posture was reconstructed, and the magnitude and spatial distribution of contact forces were visualized. In the tactile visualization, the contact force magnitude was encoded by proportionally scaled dots and a color gradient, calibrated to represent forces ranging from 0 to approximately 20 N.

#### Experimental protocol for online tool operations

**Fig. 4.**
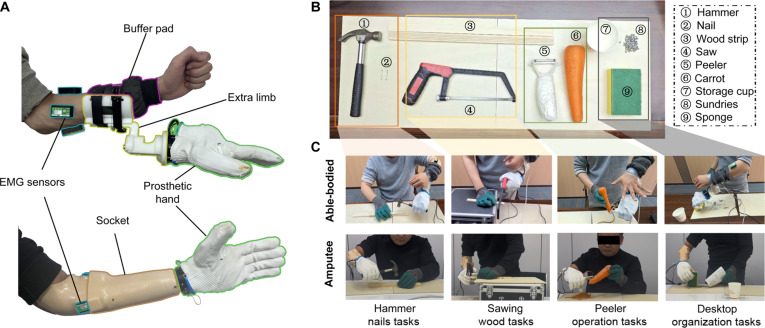
Tool-operation experimental setup. (A) Prosthetic hand wearing for able-bodied and amputee participants. (B) Tools used in the experiment. (C) Four tool-operation task demonstrations by able-bodied and amputee participants.

Before the tool-operation experiments, the prosthetic hand setup is shown in Fig. 4A. To ensure the rigor of the results and minimize learning effects, each participant underwent a standardized 25-min training session prior to formal data collection. This included a 10-min gesture mapping exercise to achieve stable control of the 5 fundamental gestures (WE, WF, HG, HO, and WS), followed by a 15-min task-specific practice where participants were required to successfully complete each of the 4 tool-operation tasks at least once under supervision. For able-bodied participants, a 3-dimensional printed extra limb was attached to their intact arm to simulate an upper-limb deficiency. For amputee participants, the prosthetic hand was connected via a customized socket. To reduce the risk of damage to the prosthetic hand during the experiments, an additional protective glove was worn. Details regarding the overall real-time operating frequencies of the system are provided in Note S1.

To effectively simulate real-world tool usage scenarios for upper-limb amputees, 4 tool-operation tasks were designed, with the corresponding tools shown in Fig. 4B. The tasks included 2 seen tasks, hammer nails and sawing wood, and 2 unseen tasks, peeler operation and desktop organization, as illustrated in Fig. 4C. These tasks were selected from common daily tool use and activities, balancing typicality and diversity and effectively reflecting the practical challenges faced by amputees during tool manipulation. Detailed descriptions of the equipment specifications, environmental constraints, and standardized procedural requirements for each task are provided in Note S2. Fig. A5 shows the operational flowchart.

Each participant was required to complete all 4 tool-operation tasks, and for each task, 3 different control methods were tested. The online operation can be found in Movie S3. Each method was repeated 5 times. To eliminate order effects, the presentation sequence of tasks and control methods was randomized. In total, 540 valid data samples were collected (9 participants × 4 tasks × 3 methods × 5 repetitions).

To quantify the temporal similarity between the human-hand demonstration data and the test data collected from able-bodied and amputee participants, this study uses dynamic time warping (DTW). DTW is well suited for evaluating sequence similarity in cases where temporal stretching or delay may occur [45]. In this work, the DTW distance and the average similarity are used as quantitative metrics: A DTW distance closer to 0 and an average similarity closer to 1 indicate a higher degree of temporal alignment between 2 signals.

The analysis results (Fig. A7) show that all comparison groups exhibit low DTW distances and high average similarity values, demonstrating pronounced temporal pattern similarity across datasets. Specifically, the “human-hand versus able-bodied” and “human-hand versus amputee” groups (Fig. A7A and B) both show relatively high similarity (approximately 0.8). This suggests that able-bodied participants and amputees can generate temporally consistent muscle activation patterns when operating the extended limb or prosthesis. Furthermore, the “able-bodied versus amputee” comparison (Fig. A7C) indicates that the EMG signals remain highly similar even when different prosthesis-wearing modalities are involved.

Overall, the combined DTW distance and similarity metrics confirm that the EMG signals used in this study share consistent core temporal patterns, supporting the validity and reliability of the experimental data.

#### Performance metrics for tool manipulation

**Fig. 5.**
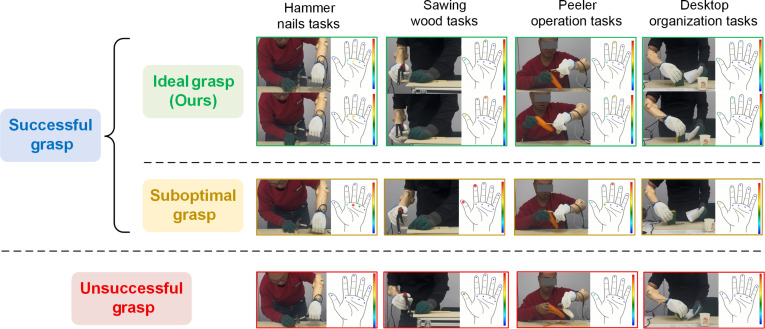
Final states of ideal, suboptimal, and unsuccessful tool-operation trials.

This study proposes that an ideal tool-operation strategy should dynamically adjust joint movements to achieve proper force balance, providing sufficient friction for each object to counteract gravity while preventing excessive deformation or potential damage. The ideal grasp and failure scenarios are illustrated in Fig. 5, which are quantitatively classified on the basis of specific contact force thresholds: An unsuccessful grasp occurs when all contact forces drop to ≤0.1 N (indicating a tool drop); a suboptimal grasp occurs when any localized force exceeds 15 N (indicating excessive mechanical load); and an ideal grasp maintains active contact forces within the optimal range of 0.1 to 15 N. For tool-operation tasks, the following evaluation metrics are defined (detailed mathematical formulations for these metrics are provided in Note S3):

• Root mean square error (RMSE): Used during offline prediction to evaluate the deviation between the tool’s predicted motion trajectory and the demonstrator’s trajectory. A smaller RMSE indicates a higher degree of anthropomorphism, operational stability, and consistency.
• Number of tool drops ($N_{\text{drop}}$): Directly evaluates the robustness and reliability of the grasping system under external perturbations by counting the number of times the tool completely slips from the hand within a single trial.
• Task completion time ($T_{\text{total}}$): Measures the overall efficiency and motion planning quality, representing the total time elapsed from the issuance of the task command to the successful placement of the tool.
• Average contact force ($F_{\mathrm{Av}}$): Evaluates the average mechanical load exerted between the tool and the end-effector. Smaller values indicate more efficient force modulation.
• Integrated EMG (iEMG): Quantifies the overall physiological work and accumulated muscle fatigue by computing the time-domain integral of the EMG signal amplitude throughout the task.
• Average EMG (AvEMG): Evaluates the overall activation intensity of the primary muscles. By analyzing the mean amplitude of EMG signals over the task duration, the muscular energy expenditure associated with the motor control strategy can be indirectly inferred.
• Subjective user ratings: To assess the usability of each control method, the USE (Usefulness, Satisfaction, and Ease of use) questionnaire [46] was administered. The USE questionnaire is a comprehensive evaluation tool that focuses on user experience and consists of 4 dimensions: usefulness, ease of use, ease of learning, and satisfaction.

#### Baseline approaches

To demonstrate the superiority of the proposed method, we compared it with existing prosthetic operation strategies. Based on differences in control strategies, current methods can be mainly divided into 2 categories: fixed force (FiF) and force follows (FoF), which are described as follows:

• FiF: This strategy applies a fixed preload after grasping the tool to maintain grasp stability and is the basic control approach adopted by most current prostheses [27,29]. Its advantages include a simple control structure, ease of implementation, and high reliability. However, it cannot dynamically adjust the grip force according to the actual operation task. If the preload is set too low, the tool may slip or drop because of external disturbances during operation. If set too high, it may cause damage to the prosthetic hand or the tool. To balance these advantages and limitations, appropriate preloads for different tools were calibrated in preliminary experiments.
• FoF: This strategy dynamically adjusts the prosthetic grip force based on real-time external load measurements from sensors, allowing the grasping force to follow task demands. It is commonly used in tactile prosthetic hands [23,28]. Its main advantage is adaptive response capability, enabling the system to adjust grip force at different stages of operation and improve safety. Its limitations include high dependence on sensor accuracy and response speed and potential force instability or tracking delay under dynamic or impact loads. In this study, reference target forces for different tools were set during preliminary experiments.

#### Statistics analysis

This study used one-way analysis of variance (ANOVA) to test for significant differences in performance metrics among different models, with a significance level set at 0.05.

## Results

### Offline prediction of joint motion performance analysis

The collected demonstrator data were randomly split into training, validation, and test sets with a 6:2:2 ratio. Based on the training and validation sets, the TKE-BGC model was constructed and trained. To assess its offline prediction capability, we compared 3 methods, FiF, FoF, and TKE-BGC, on the same test set.

**Fig. 6.**
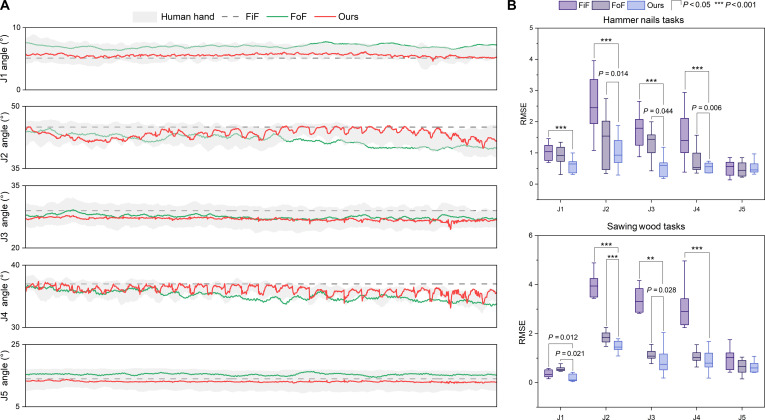
Offline prediction results of the 3 control methods. (A) Visualization of predicted joint motion curves during the hammering task (partial data). (B) Comparison of root mean square error (RMSE) values in offline operation tasks. **P < 0.01 and ***P < 0.001.

Fig. 6A shows the predicted joint torques of the 5 joints for the 3 control methods during the tool operation tasks. The FiF method lacks an online feedback adjustment mechanism. When the demonstrator manually adjusts because of external perturbations (gray curves), FiF fails to follow the actual operations and does not exhibit dynamic adaptability. The FoF method has some disturbance rejection capability and can adjust when perturbations occur, but its response shows noticeable delay, resulting in deviations between the predicted curves and the demonstration data and limited tracking accuracy. In contrast, the proposed method can rapidly adjust during continuous perturbations, with predicted curves largely remaining within the demonstration trajectories. Analysis of the RMSE metrics (Fig. 6B) shows that for the 2 offline tool operation tasks, especially for joints J2, J3, and J4, which are primarily adjusted by the demonstrator during operation, the feedback control requirements are higher compared to other joints. The proposed method achieves significantly better prediction performance than the other 2 methods, particularly in the hammering task, where rigid impacts are more pronounced.

### Performance analysis of online tool operation tasks

**Fig. 7.**
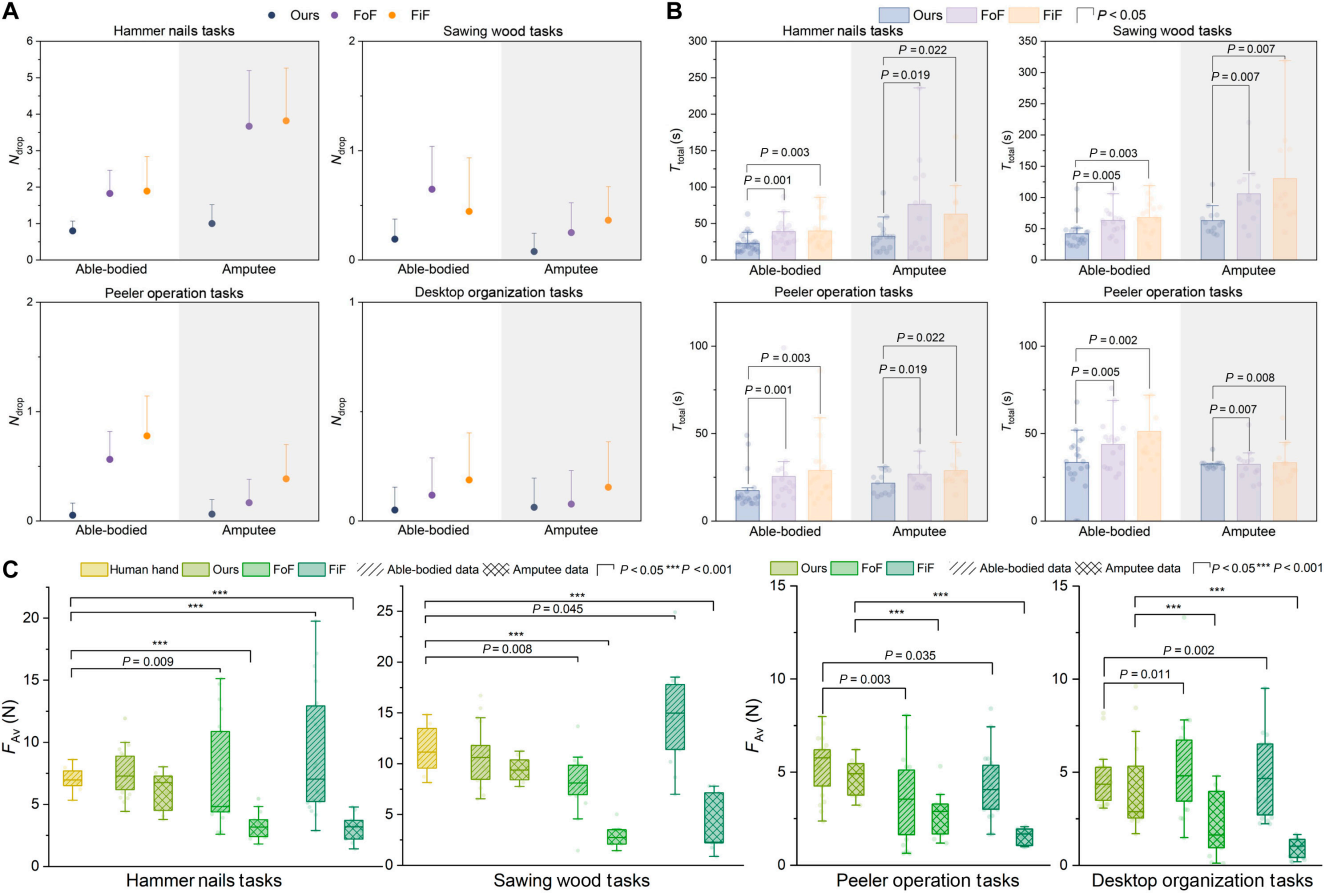
Evaluation metrics of the 3 control methods across 4 tool operation tasks. (A) Number of tool drops during task execution (means + SD). (B) Task completion time. (C) Average contact force, with demonstration data (light yellow) included as a reference baseline in the hammer nails and sawing wood tasks. Statistical significance is indicated at *P* < 0.05.

Fig. 7 presents the performance evaluation of the prosthetic hand across different control methods during the tool operation tasks. Regarding the number of tool drops (Fig. 7A, all methods exhibited higher drop counts in the hammer nails tasks due to stronger rigid impacts, but the proposed method consistently showed significantly fewer drops than FoF and FiF across all tasks. In terms of task completion time (Fig. 7B), the proposed method achieved shorter operation times in all tasks and significantly outperformed the other 2 methods. In longer-duration tasks such as the sawing wood tasks, the robust performance of Ours resulted in more pronounced time advantages. For average contact force (Fig. 7C), demonstration data were included for comparison in the hammer nails and sawing wood tasks. Ours achieved contact force distributions similar to those of the human hand, whereas FoF and FiF showed significant deviations from human performance. In the 2 unseen tasks, peeler operation and desktop organization, Ours maintained stable contact force levels for both able-bodied and amputee participants, and performed significantly better than the other methods. Movies S4 to S11 show online demonstrations comparing the performance of the 3 methods for both able-bodied and amputee participants.

### Evaluation of user operational load and experience

**Fig. 8.**
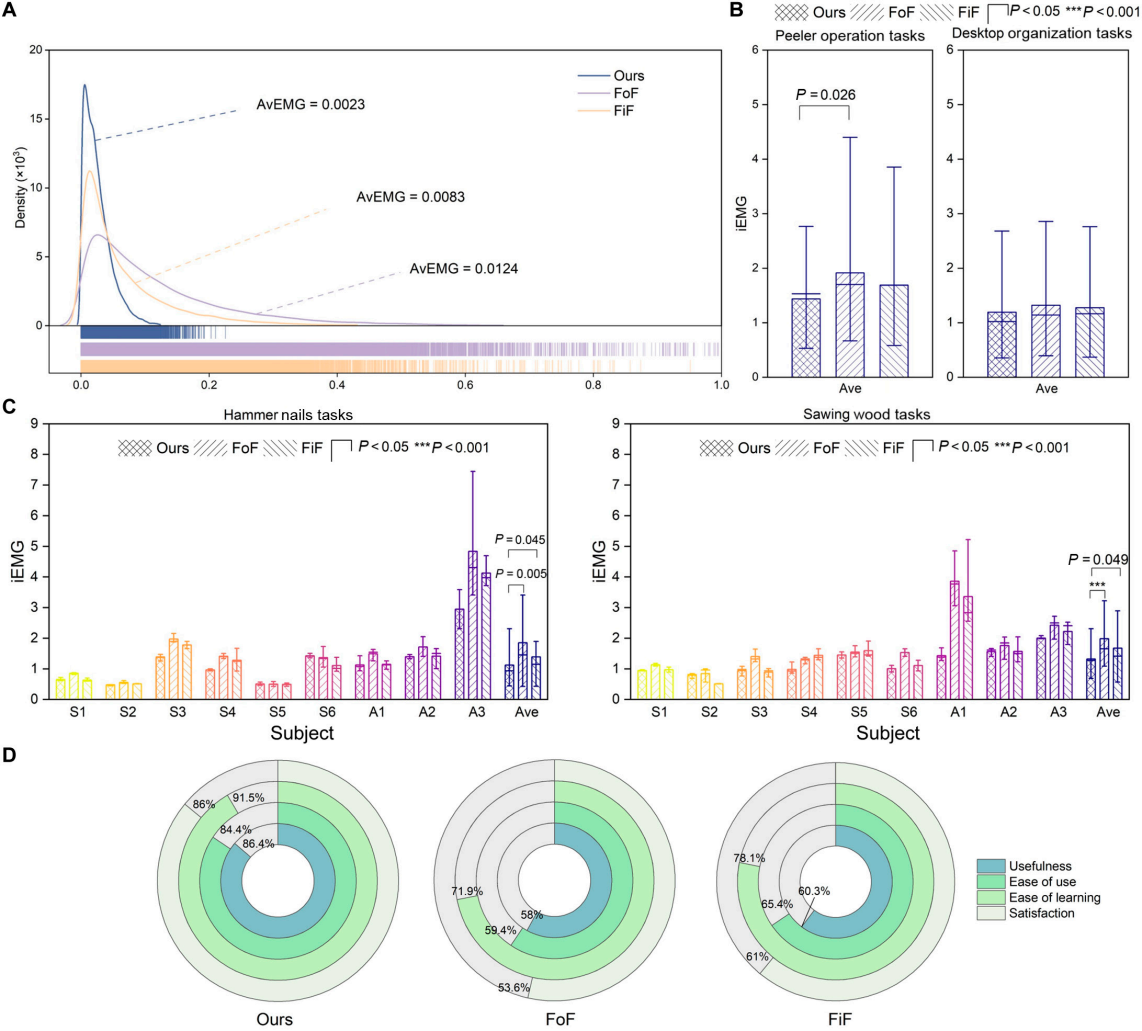
User workload and subjective evaluation results. (A) Normalized electromyography (EMG) amplitude distribution statistics from partial operation data in the hammer nails tasks using 3 control methods. Higher peaks indicate more concentrated EMG amplitude distributions, while distributions farther from the origin represent greater muscle activation levels. (B) Integrated EMG (iEMG) histograms for the 2 unseen operation tasks, where “Ave” denotes the averaged results across all participants. (C) iEMG histograms for the 2 seen operation tasks, including individual and averaged iEMG statistics. “*P* < 0.05” indicates statistically significant differences. (D) Results of the USE (Usefulness, Satisfaction, and Ease of use) questionnaire, covering 4 dimensions: usefulness, ease of use, ease of learning, and satisfaction.

Fig. 8 presents the assessment of user workload and subjective feedback for different control methods during the tool operation tasks. The distribution of EMG amplitudes reflects muscle activation during actual operation. Fig. 8A shows normalized EMG amplitude statistics from partial operation data in the hammer nails tasks for the 3 methods. The amplitude distribution for Ours is mostly concentrated in the range of 0 to 0.2, indicating lower required muscle effort compared to the other methods (AvEMG: Ours versus FoF versus FiF, 0.0023 versus 0.0124 versus 0.0083). Further analysis of iEMG, which reflects the overall muscle load during the tasks, shows that in the 2 unseen tasks (Fig. 8B), Ours exhibits lower iEMG than the other methods, although the differences are not significant, likely because these tasks involve smaller external impacts. In the 2 seen tasks (Fig. 8C), amputee participants generally have higher iEMG than able-bodied participants, possibly due to differences in prosthesis attachment. Nevertheless, the Ours method achieved the lowest iEMG for both able-bodied and amputee participants.

In the subjective user survey (Fig. 8D), the proposed method received highly positive evaluations, particularly from amputee participants, who noted that it improved operational efficiency while reducing physical effort. In addition, some amputee subjects pointed out that although the FoF method can rapidly adjust joint movements in response to external impacts, its adjustment direction often opposes the external force and lacks effective predictive capability; therefore, its overall evaluation was inferior to that of the FiF method.

### Ablation experiments

**Fig. 9.**
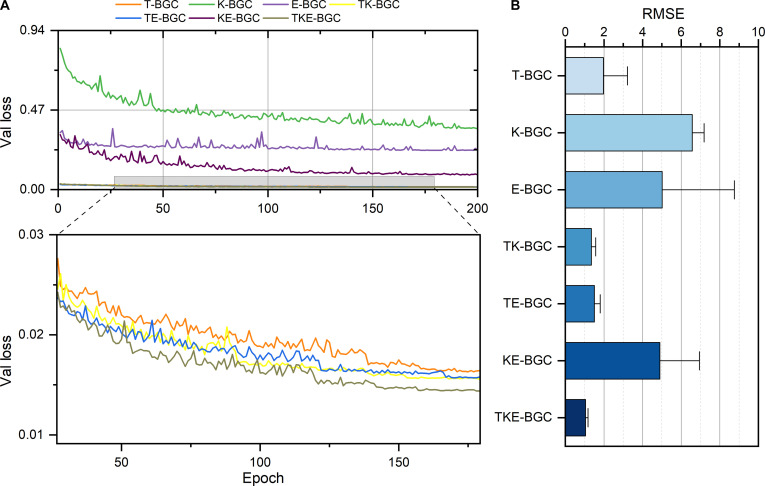
Ablation study results of the tactile, kinesthetic, and electromyography bionic gripping controller (TKE-BGC) model. (A) Validation loss (Val loss) obtained by sequentially removing each input modality from the model. (B) Performance comparison of the ablated models on the hammer nails and sawing wood tasks, evaluated using the root mean square error (RMSE) between predicted and ground-truth actions. The average RMSE across both tasks was used for overall comparison.

We conducted an ablation study on the TKE-BGC model to evaluate the contributions of tactile (T), kinesthetic (K), and EMG (E) modalities. The results are shown in Fig. 9. It can be observed that tactile features play a critical role in tool operation. Specifically, compared to models using only kinesthetic features (K-BGC) or only EMG features (E-BGC), the model using only tactile features (T-BGC) achieved better performance in terms of validation loss and RMSE, indicating that tactile information significantly improves prediction accuracy. Furthermore, combining multiple modalities led to continuous performance improvement. Adding EMG or kinesthetic features to the tactile-based model further enhanced predictive capability. Finally, when all 3 modalities were combined as input (TKE-BGC), the controller achieved the best prediction performance, confirming the advantages of multimodal fusion.

## Discussion

### The role of tactile feedback in manipulation

Tactile perception plays a crucial role in intelligent prosthetic hand operation, especially when visual feedback is unavailable. Our method relies entirely on the user’s real-time proprioceptive input and does not require any prior knowledge or visual observation to achieve efficient operation. It ensures effective contact force management and finger coordination, resulting in performance closely resembling human hand grasping.

As shown in Fig. 9, the T-BGC model achieves a certain level of prediction accuracy, indicating that tactile perception has a more significant impact compared to kinesthetic and EMG modalities. With the inclusion of additional input modalities, the controller’s feedback accuracy improves significantly. EMG signals provide real-time feedback on the user’s current state and intentions, and adding the EMG modality substantially enhances predictive performance. Kinesthetic information contributes historical joint movement data of the dexterous hand, further improving prediction accuracy. However, our method uses a reduced yet rational tactile representation (Fig. 3C), emphasizing the key role of low-dimensional full-hand tactile feedback in grasping. In comparison, human tactile perception is denser and includes additional capabilities, such as object temperature sensing and lateral shear strain detection, which are essential for more complex intelligent prosthetic hand operations.

### The generalizability and robustness of our approach

This study systematically evaluated the generalizability and robustness of the proposed method across 3 dimensions: subject generalization, task generalization, and prosthesis adaptability.

Regarding subject generalization, the proposed method can extract universal, physics-based motor strategies from the demonstrations of a single participant and achieve consistent and superior performance across multiple able-bodied and amputee participants within the scope of the tested tool-use scenarios. In the tool operation tasks (Figs. 7 and 8), all participants using TKE-BGC demonstrated low tool drop rates, contact force levels comparable to the human hand, and stable task completion times. In addition, iEMG values were lower than those of comparative methods, indicating that the system can effectively adapt to different users’ EMG patterns and possesses strong cross-subject adaptability.

For task generalization, TKE-BGC not only performed excellently on seen training tasks, such as hammer nails and sawing wood but also maintained high operational stability on unseen tasks, including peeler operation and desktop organization (Figs. 7 and 8B). These results suggest that the learned grasping and manipulation strategies can transfer effectively to new tools and scenarios, demonstrating substantial task generalization capability. Furthermore, the proposed method exhibits good bilateral prosthesis compatibility. Through a unified multimodal control framework, demonstration data collected from the right hand can be directly applied to left-hand operation, achieving symmetric performance without additional adjustments. As shown in Fig. 8C, left-hand amputees (A1 and A3) achieved comparable performance to a right-hand amputee (A2).

Finally, robust grasping control enabled participants to perform tool operations more stably and with less effort (Fig. 8A to C) and elicited positive subjective feedback (Fig. 8D). Overall, TKE-BGC constructs a robust and generalizable prosthetic control framework by effectively integrating tactile, kinesthetic, and EMG information. Its performance advantage stems from the transfer of human hand manipulation skills: Tactile information enables sensitive contact force regulation, kinesthetic features ensure continuous hand motion, and EMG signals accurately reflect user intent. The synergy of these multimodal inputs allows the prosthesis to achieve reliable and human-like operation in realistic and dynamic scenarios.

### Limitations and future works

This study focused on prosthetic hand tool operation tasks and explored the potential of prostheses in tool-use scenarios by integrating the user’s proprioceptive feedback, multimodal information fusion, and the transfer of human hand skills from demonstrators. However, several limitations remain.

First, the data glove used in the experiments provided only sparse tactile sampling points and could measure only contact force. Such sparse force-based feedback cannot fully replicate the rich tactile experiences of human operation, including vibration, temperature, deformation, and lateral shear strain. Nevertheless, experimental results showed that even limited tactile features can significantly improve operational performance. In the future, higher-density, miniaturized, and modular tactile sensing systems could be developed to more closely approximate real tactile perception. We plan to incorporate advanced high-density tactile sensors and process local tactile images with neural networks to capture finer spatial interactions during operation.

Second, for dynamic operation tasks, finger abduction movements were not modeled in this study to improve training efficiency and system robustness. However, in more complex multi-DoF tasks, fine-grained motion representation may offer advantages. Future work will consider more comprehensive motion representation strategies to support dexterous manipulation.

Third, relying on a single demonstrator constitutes a single-source imitation setting. The successful cross-population transfer relies on shared physical constraints in dynamic tasks, yielding a standardized stabilization strategy. However, this setup lacks personalized grasping preferences. Thus, our current conclusions are limited to transferring fundamental physical stability. Future work will utilize multisource datasets from diverse participants to capture personalized manipulation styles.

Finally, the TKE-BGC output was defined as joint angle increments, which simplified the training process and facilitated rapid transfer across different prosthetic platforms without the need for data recollection or retraining. However, the current mapping still relies on manual parameter adjustment. Our next step is to introduce an optimization-based mapping method to achieve adaptive alignment of individual finger postures, thereby improving the intelligence and adaptability of prosthetic control.

## Conclusion

This study proposes an EMG-based prosthetic control framework based on human skill transfer. It enables complete control from EMG PR to tool grasping and manipulation. The framework can generate human-like contact force distributions during operation, effectively reducing excessive force while significantly improving the stability and reliability of grasping and manipulation. With its robust grasping performance, users can complete tasks efficiently with less effort, resulting in reduced fatigue and more natural control. Systematic experiments on 4 tool operation tasks, including both seen and unseen scenarios, demonstrate that the proposed framework possesses strong robustness and task generalization capability. The findings of this study hold practical significance for enhancing the daily living independence of upper-limb amputees and facilitating their vocational rehabilitation and reemployment.

## Data Availability

The experimental subject operation dataset can be provided by B.F. pending scientific review and a completed material transfer agreement. Requests for the dataset should be submitted to B.F.

## Supplementary Materials

Supplementary 1 Notes S1 to S3 Figs. A1 to A7 Tables A1 to A3 Movies S1 to S11
