# Supplementary material for: Dynamic Manipulation Skill Learning for Tactile Myoelectric Prosthetic Hands in Tool Handling
Source: Cyborg Bionic Syst. 2026 May 13;7:0572. doi: 10.34133/cbsystems.0572 (PMC13168764; doi:10.34133/cbsystems.0572)
Supplement: Supplementary 1 — Notes S1 to S3 Figs. A1 to A7 Tables A1 to A3 Movies S1 to S11 [file cbsystems.0572.f1.zip › Supplementary Materials.docx]

SUPPLEMENTARY MATERIALS

**Supplementary Note S1:** System Deployability and Real-Time Performance

To validate the practical deployability of the framework, real-time performance metrics were systematically evaluated. The average inference latency of the TKE-BGC model is approximately 38 ms on the local computational unit. As illustrated in Fig. A5, the entire myoelectric bionic prosthetic control system operates as a closed loop with varying module frequencies. While the low-level prosthetic hand executes commands at 100 Hz, the TKE-BGC controller processes multimodal feedback and updates control strategies at 20-30 Hz. Considering the 15-20 Hz online intent recognition, the overall closed-loop system achieves a stable operation frequency of 10-15 Hz. This corresponds to an overall system latency of approximately 66-100 ms, which is significantly lower than the standard human sensorimotor response time, ensuring smooth and responsive continuous tool operation for the amputee users.

**Supplementary Note S2:** Detailed Protocols for Online Tool Operations

Throughout data collection, the prosthetic hand was constrained within a predefined operational area, and the wrist posture was kept relatively fixed. Before each task, relevant objects were placed within reach of the participants. Contact force data were zero-drift calibrated prior to acquisition, with recordings starting when the prosthetic hand was fully open. The following sections provide detailed descriptions of the procedures and requirements for the four tool-operation tasks.

**Hammer Nails Tasks:** Participants used the prosthetic hand to grasp a hammer and drive a standard nail into two stacked pine boards until the nail penetrated both layers and securely connected them. If the hammer slipped or was dropped due to reaction forces during striking, participants were required to re-grasp the tool and continue from the previous operation until the task was completed. The task was considered successful when the two boards were firmly connected and did not wobble or fall.

**Sawing Wood Tasks:** Participants held a hand saw with the prosthetic hand to perform transverse cutting on pine strips with a cross-section of 20mm × 10mm. The goal was to completely sever the wood strip. If the saw slipped or was dropped due to resistance or posture changes during cutting, participants needed to re-establish a stable grasp and continue cutting from the existing kerf.

**Peeler Operation Tasks:** Participants used the prosthetic hand to operate a peeler on a fresh carrot approximately 120mm in length and 25mm in diameter. The task required peeling along the longitudinal axis of the carrot evenly, producing six continuous strips of uniform width. If the tool slipped or was dropped during peeling, participants were allowed to re-grasp the peeler and continue.

**Desktop Organization Tasks:** Participants used the prosthetic hand to grasp sponge blocks and collect scattered items on a desk, stacking them in a designated area. They then used an A4-sized rigid sheet to scoop the items and transfer them into a storage cup. If the tools slipped during pushing or scooping, participants could re-grasp and continue. The task was deemed successful when all items were transferred into the cup without remaining in the workspace.

**Supplementary Note S3:** Mathematical Formulations of Performance Metrics

The quantitative performance metrics utilized in this study are mathematically defined as follows:

**1. Root Mean Square Error (**RMSE**):**

$$\text{RMSE}=\sqrt{\frac{1}{n}\sum_{i=1}^{n} \left( y_{i}-\hat{y}_{i} \right)^{2}}$$

where $y_{i}$ denotes the actual value, $\hat{y}_{i}$ denotes the predicted value, and $n$ is the number of data points.

**2. Task Completion Time (**$T_{\text{total}}$**):**

$$T_{\text{total}}=t_{\text{end}}-t_{\text{start}}$$

where $t_{\text{start}}$ denotes the start time of the participant’s tool operation, and $t_{\text{end}}$ denotes the end time when the tool operation task is completed and the prosthetic hand is fully opened.

**3. Average Contact Force (**$\bar{F}$**):**

$$\bar{F}=\frac{1}{t_{\text{end}}-t_{\text{start}}}\int_{t_{\text{start}}}^{t_{\text{end}}} \left\| \vec{F}\left( t \right) \right\|dt$$

where $\vec{F}\left( t \right)$ represents the total contact force vector measured between the tool and the end-effector at time $t$, and $\left\| \vec{F}\left( t \right) \right\|$ denotes its magnitude.

**4. Integrated EMG (**iEMG**):**

$$\text{iEMG}=\int_{0}^{T} \left| \text{EMG}\left( t \right) \right|dt$$

where $\left| \text{EMG}\left( t \right) \right|$ denotes the processed EMG signal amplitude, and $T$ is the total task duration.

**5. Average EMG (**AvEMG**):**

The mean of the full-wave rectified signals is computed as the average EMG amplitude for each muscle in the task. The overall metric is expressed as the mean of the EMG values across the target muscles:

$$\text{AvEMG}=\frac{1}{N}\sum_{m=1}^{N} \left( \frac{1}{T}\int_{0}^{T} \left| \text{EM}\text{G}_{\text{m}}\left( t \right) \right|dt \right)$$

where $N$ denotes the number of EMG channels, $T$ is the total task duration, and $\text{EM}\text{G}_{\text{m}}\left( t \right)$ represents the processed EMG value of the $\text{m}$-th channel at time $t$.

Fig. A1. DCNN-GRU architecture for EMG-based gesture recognition. The input EMG feature maps are processed by two inception block to produce feature maps, which are recalibrated via parallel Squeeze-and-Excitation (SE) and spatial attention (SA) modules and fused residually. The fused feature maps are down sampled by max pooling and subsequently input into multi-layer GRU to capture temporal correlations. Finally, a fully connected layer and Softmax classifier output the gesture recognition results.

Fig. A2. Network structure of multi-head attention mechanism.

Fig. A3. Decoding of gesture recognition in human subjects. Representative real-time predictions and EMG signals from the first able-bodied (a) and amputee (b) subjects. For clarity and convenience, all six hand gestures used for control are shown across the panels. (c) Classification confusion matrices for both groups of subjects.

Fig. A4. Demonstration data of two tool-operation tasks. Visualization of (a) the Hammer Nails and (b) the Sawing Wood task.

Fig. A5. Integration framework and operational frequencies of the myoelectric bionic prosthetic hand control system.

Fig. A6. Flowchart of the four types of tool-operation tasks.

Fig. A7. The similarity analysis across different participant groups is presented from left to right:(a) human-hand data versus able-bodied data, (b) human-hand data versus amputee data, and (c) able-bodied data versus amputee data. For each comparison, both the DTW distance and the average similarity are reported, with all results computed as the mean across multiple datasets.

Table A1. Network structure parameters of DCNN.

Table A2. Training hyperparameters and setup.

Table A3. Clinical characteristics of the amputee participants.

Movie S1. Data collection process for a nail-hammering task. Animated version of Figure A4(a).

Movie S2. Data collection process for a sawing task. Animated version of Figure A4(b).

Movie S3. Operational task demonstrations by amputee subjects.

Movie S4. A temporal comparison of three approaches used by able-bodied subjects in the Hammer Nails Task. The demo videos shown are taken from the fastest-performing group for each approach.

Movie S5. A temporal comparison of three approaches used by able-bodied subjects in the Sawing Wood Task. The demo videos shown are taken from the fastest-performing group for each approach.

Movie S6. A temporal comparison of three approaches used by able-bodied subjects in the Peeler Operation Task. The demo videos shown are taken from the fastest-performing group for each approach.

Movie S7. A temporal comparison of three approaches used by able-bodied subjects in the Desktop Organization Task. The demo videos shown are taken from the fastest-performing group for each approach.

Movie S8. A temporal comparison of three approaches used by amputee subjects in the Hammer Nails Task. The demo videos shown are taken from the fastest-performing group for each approach.

Movie S9. A temporal comparison of three approaches used by amputee subjects in the Sawing Wood Task. The demo videos shown are taken from the fastest-performing group for each approach.

Movie S10. A temporal comparison of three approaches used by amputee subjects in the Peeler Operation Task. The demo videos shown are taken from the fastest-performing group for each approach.

Movie S11. A temporal comparison of three approaches used by amputee subjects in the Desktop Organization Task. The demo videos shown are taken from the fastest-performing group for each approach.


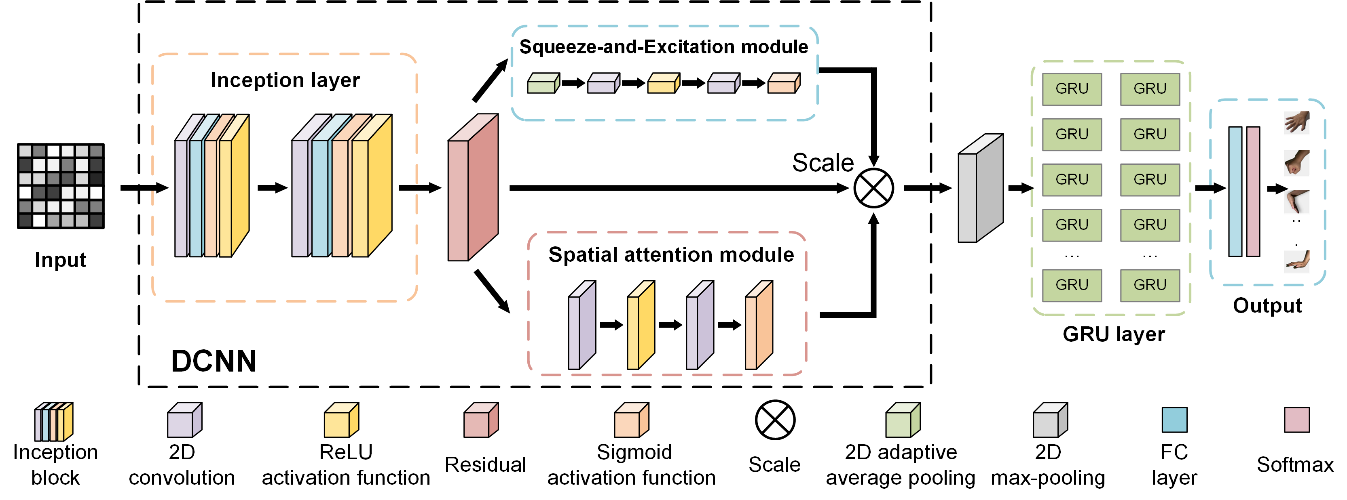


Figure A1. DCNN-GRU architecture for EMG-based gesture recognition. The input EMG feature maps are processed by two inception block to produce feature maps, which are recalibrated via parallel Squeeze-and-Excitation (SE) and spatial attention (SA) modules and fused residually. The fused feature maps are down sampled by max pooling and subsequently input into multi-layer GRU to capture temporal correlations. Finally, a fully connected layer and Softmax classifier output the gesture recognition results.


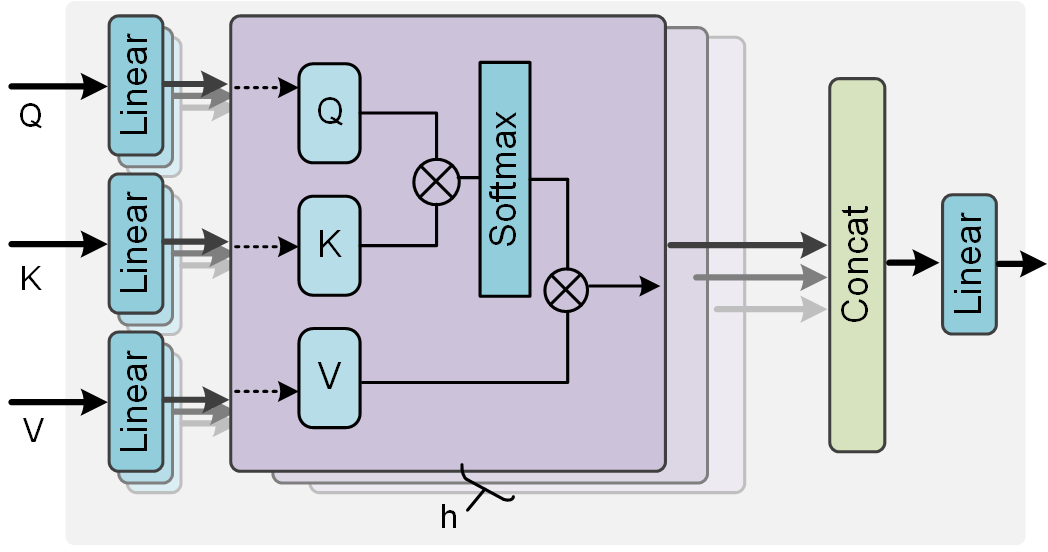


**Figure A2: Network structure of multi-head attention mechanism.**


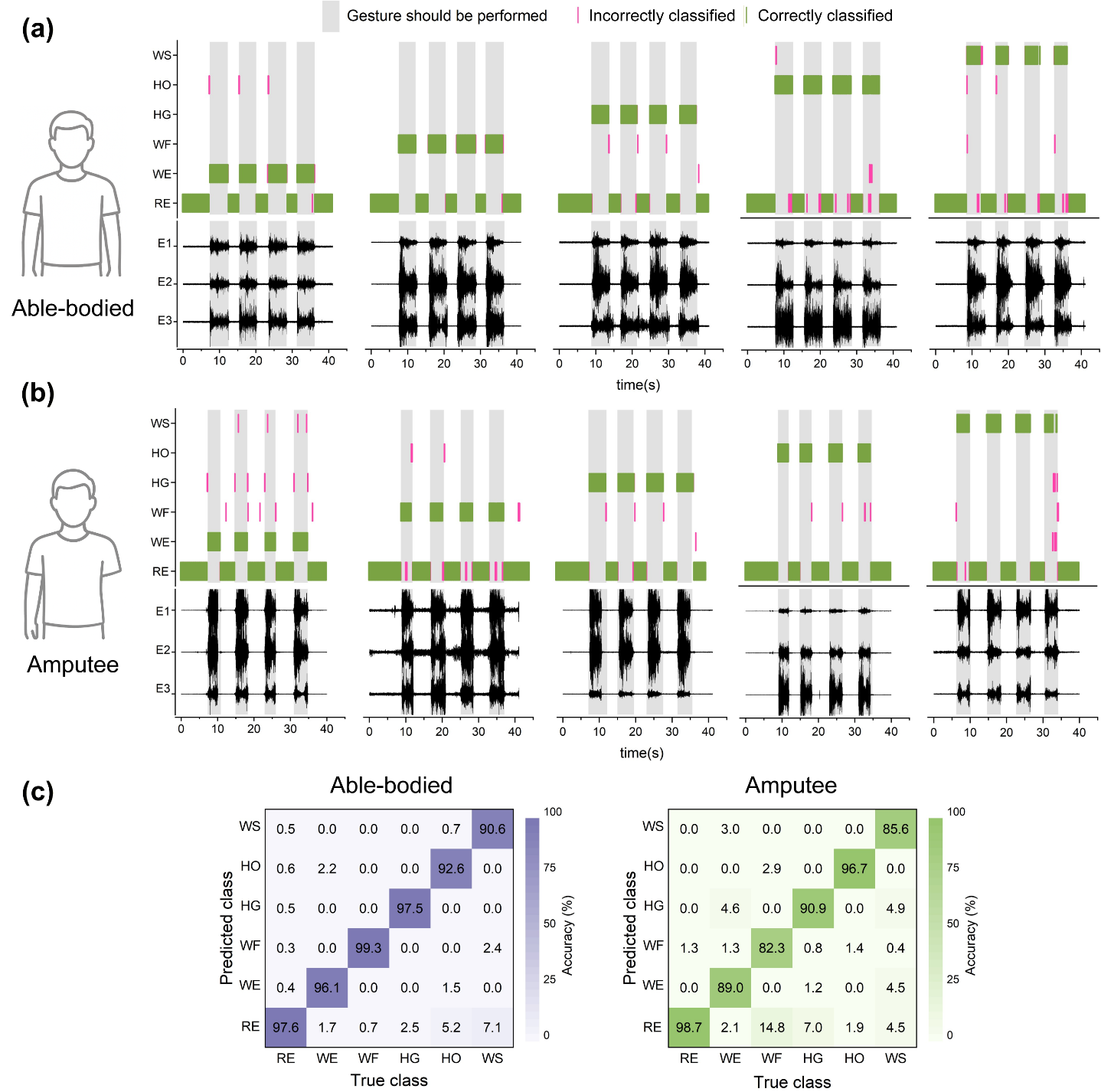


**Figure A3: Decoding of gesture recognition in human subjects.** Representative real-time predictions and EMG signals from the first able-bodied (a) and amputee (b) subjects. For clarity and convenience, all six hand gestures used for control are shown across the panels. (c) Classification confusion matrices for both groups of subjects.


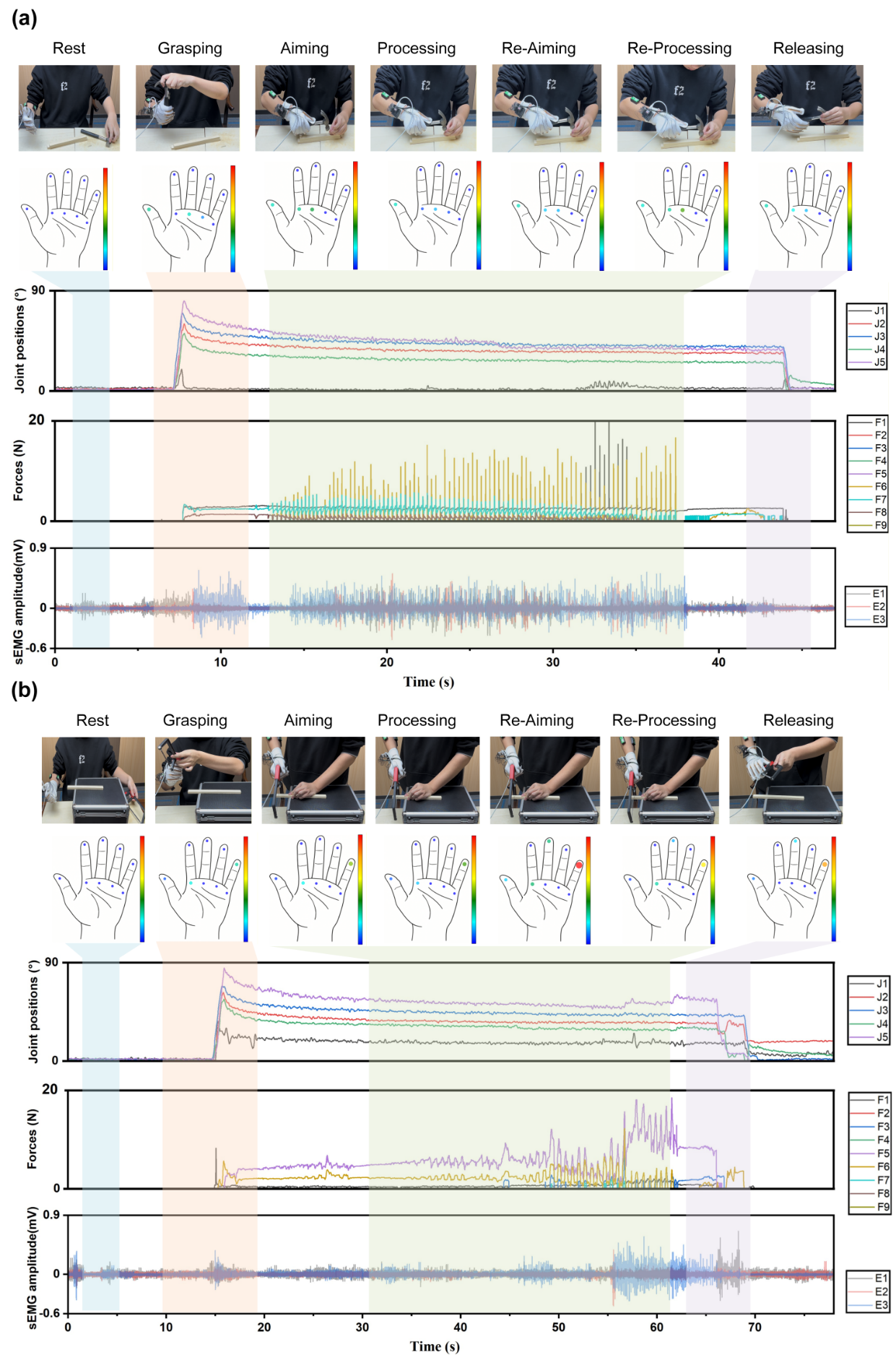


**Figure A4: Demonstration data of two tool-operation tasks.** Visualization of (a) the Hammer Nails and (b) the Sawing Wood task.


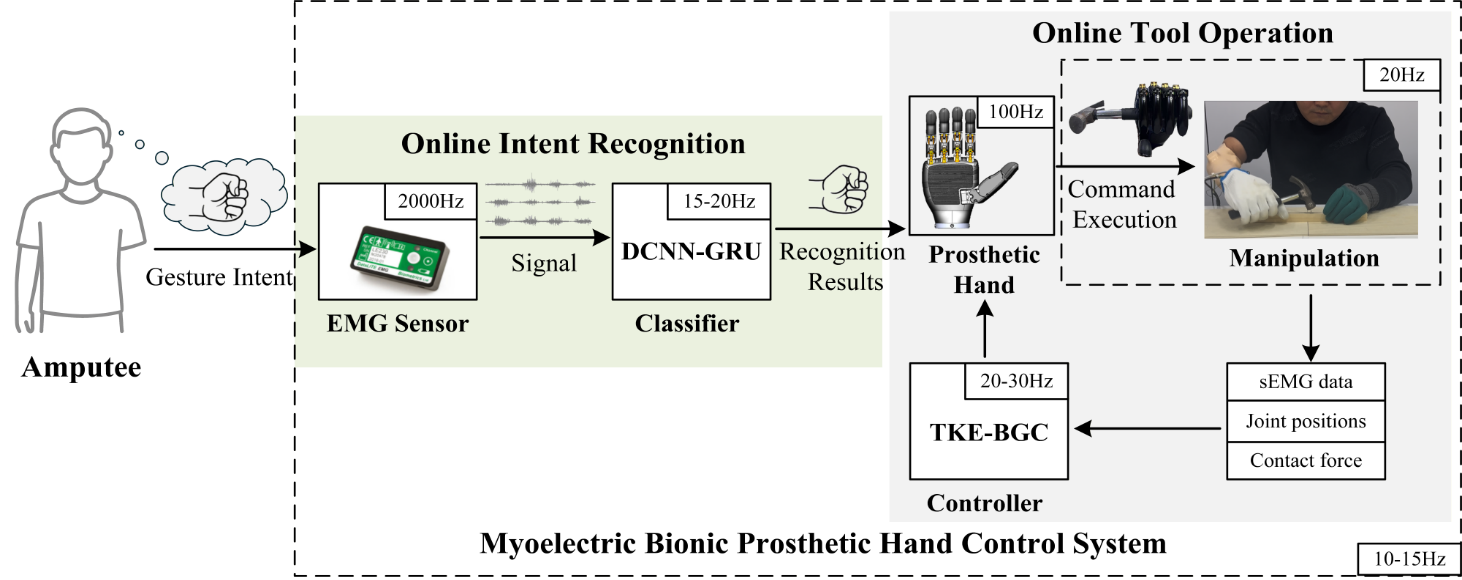


**Figure A5: Integration framework and operational frequencies of the myoelectric bionic prosthetic hand control system.**


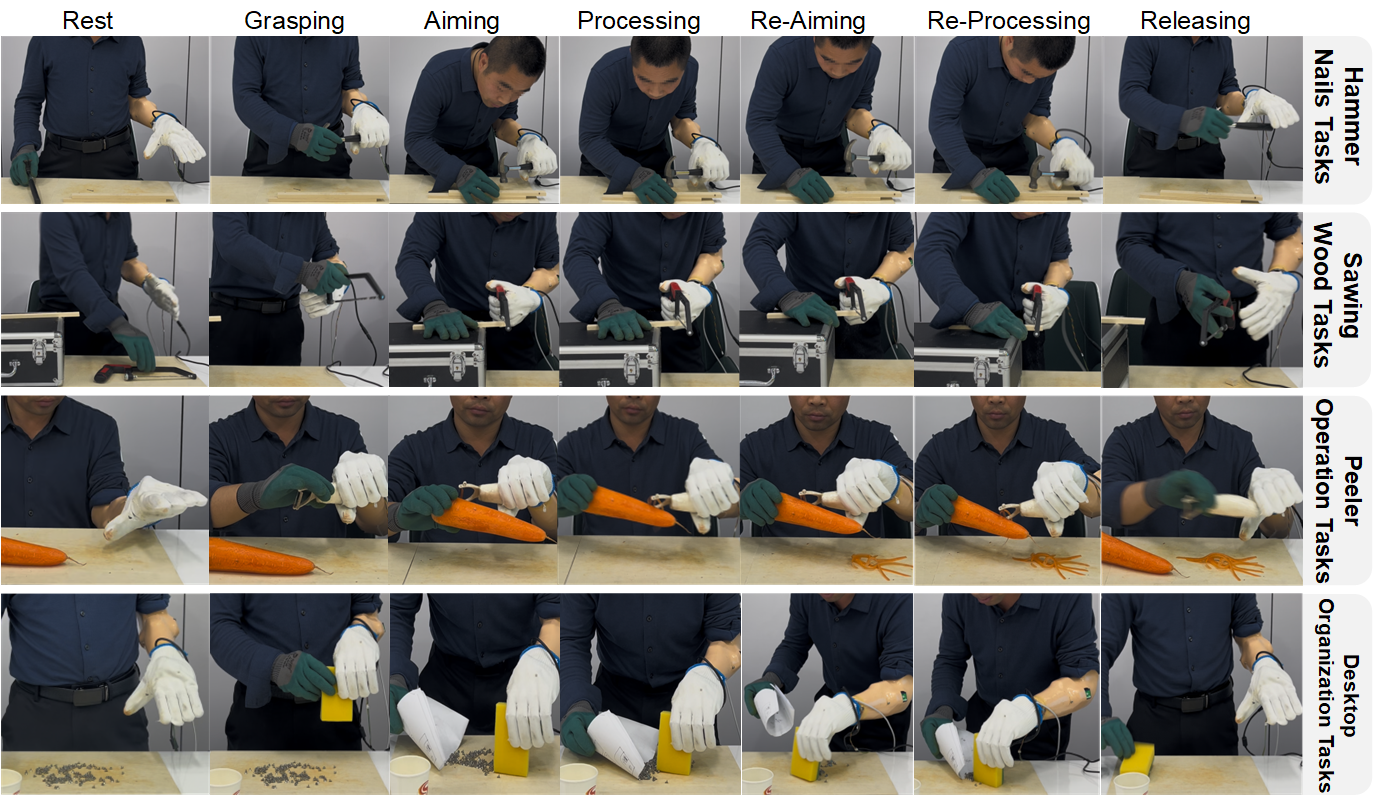


**Figure A6: Flowchart of the four types of tool-operation tasks.**

**
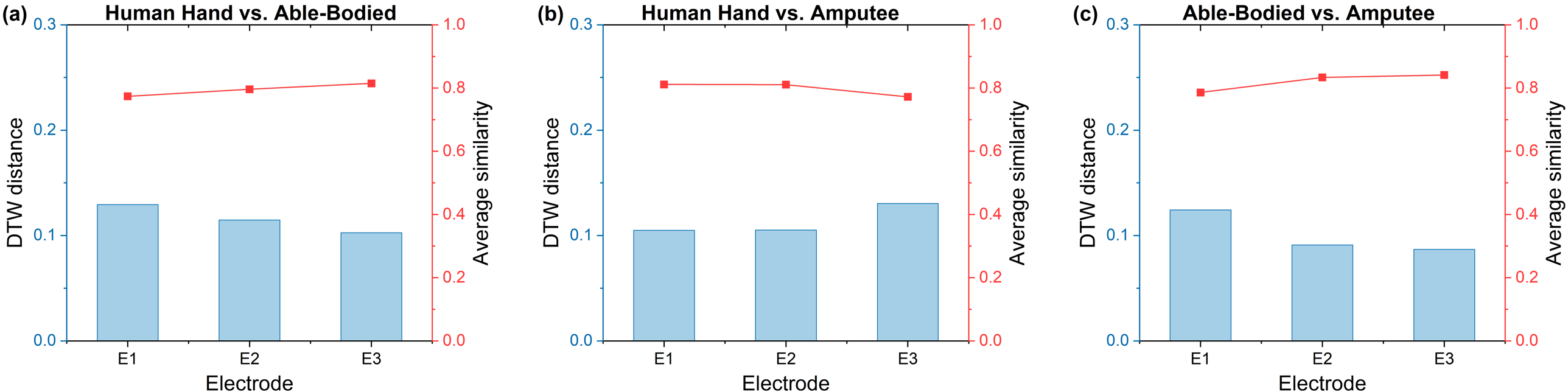
**

**Figure A7: The similarity analysis across different participant groups is presented from left to right:**(a) human-hand data versus able-bodied data, (b) human-hand data versus amputee data, and (c) able-bodied data versus amputee data. For each comparison, both the DTW distance and the average similarity are reported, with all results computed as the mean across multiple datasets.

***Table A1****. Network structure parameters of DCNN.*

| **Layer** | **Compositions** | **k** | **s** | **p** | **In/Out** |
| --- | --- | --- | --- | --- | --- |
| Branch1 | Conv+BN+ReLU | 1 | 1 | 0 | 1/8 |
| Branch2 | Conv+BN+ReLU | 1 | 1 | 0 | 1/8 |
|  | Conv+BN+ReLU | 3 | 1 | 1 | 8/8 |
| Branch3 | Conv+BN+ReLU | 1 | 1 | 0 | 1/8 |
|  | Conv+BN+ReLU | 5 | 1 | 2 | 8/8 |
| Branch4 | MaxPool | 3 | 1 | 1 | 1/1 |
|  | Conv+BN+ReLU | 1 | 1 | 0 | 1/8 |
| Concatenate | - | - | - | - | 8+8+8+8/32 |
| Branch5 | Conv+BN+ReLU | 1 | 1 | 0 | 32/16 |
| Branch6 | Conv+BN+ReLU | 1 | 1 | 1 | 32/16 |
|  | Conv+BN+ReLU | 3 | 1 | 1 | 16/16 |
| Branch7 | Conv+BN+ReLU | 1 | 1 | 1 | 32/16 |
|  | Conv+BN+ReLU | 5 | 1 | 2 | 16/16 |
| Branch8 | MaxPool | 3 | 1 | 1 | 32/32 |
|  | Conv+BN+ReLU | 1 | 1 | 1 | 32/16 |
| Concatenate | - | - | - | - | 16+16+16+16/64 |
| Residual | - | - | - | - | 64/64 |
| SE | AvgPool | - | - | - | 64/64 |
|  | Conv+ReLU | 1 | 1 | 0 | 64/16 |
|  | Conv+Sigmoid | 1 | 1 | 0 | 16/64 |
| SA | Conv+ReLU | 3 | 1 | 1 | 64/16 |
|  | Conv+ Sigmoid | 3 | 1 | 1 | 16/64 |

(Where Conv, BN, ReLU, MaxPool, AvgPool, and Sigmoid denote 2D convolution, batch normalization, ReLU activation function, Max pooling, 2D adaptive average pooling, and Sigmoid activation function, respectively, k, s, and p represent kernel size, stride, and padding, respectively, Residual refers to the operation of preserving original features and integrating high-order features through skip connections.)

***Table A2****. Detailed architectural specifications and training hyperparameters.*

| Category | Parameter | Value |
| --- | --- | --- |
| Input | *P* | 20 |
| Feature Extraction (CNN) | CNN Channels | [32, 64] |
|  | Kernel Size | 3 |
| Multi-Head Attention | Layers | 1 |
|  | *h* | 4 |
|  | *d_k_* | 64 |
|  | *d_a_* | 16 |
|  | Dropout Rate | 0.1 |
| Prediction Network (MLP) | Hidden Layers | 3 |
|  | Hidden Dims | [256, 128, 64] |
|  | Activation | ReLU |
| Training Settings | Optimizer | Adam |
|  | Learning Rate | 1e-3 |
|  | Batch Size | 128 |
|  | Epoch | 200 |
|  | Loss Function | MSE |

***TableA 3****. Detailed architectural specifications and training hyperparameters.*

| Subject | Amputated hand | Years since amputation | Prosthesis use |
| --- | --- | --- | --- |
| A1 | Left | 3.5 | No |
| A2 | Right | 29.0 | Cosmetic |
| A3 | Left | 8.0 | Myoelectric |
